# Specific neurophysiological mechanisms underlie cognitive inflexibility in inflammatory bowel disease

**DOI:** 10.1038/s41598-017-14345-5

**Published:** 2017-10-24

**Authors:** Vanessa A Petruo, Sebastian Zeißig, Renate Schmelz, Jochen Hampe, Christian Beste

**Affiliations:** 1 Cognitive Neurophysiology, Department of Child and Adolescent Psychiatry, Faculty of Medicine of the TU Dresden, Dresden, Germany; 2 Department of Internal Medicine I, University Hospital Dresden, Faculty of Medicine of the TU Dresden, Dresden, Germany; 3 Experimental Neurobiology, National Institute of Mental Health, Klecany, Czech Republic

## Abstract

Inflammatory bowel disease (IBD) is highly prevalent. While the pathophysiological mechanisms of IBD are increasingly understood, there is a lack of knowledge concerning cognitive dysfunctions in IBD. This is all the more the case concerning the underlying neurophysiological mechanisms. In the current study we focus on possible dysfunctions of cognitive flexibility (task switching) processes in IBD patients using a system neurophysiological approach combining event-related potential (ERP) recordings with source localization analyses. We show that there are task switching deficits (i.e. increased switch costs) in IBD patients. The neurophysiological data show that even though the pathophysiology of IBD is diverse and wide-spread, only specific cognitive subprocesses are altered: There was a selective dysfunction at the response selection level (N2 ERP) associated with functional alterations in the anterior cingulate cortex and the right inferior frontal gyrus. Attentional selection processes (N1 ERP), perceptual categorization processes (P1 ERP), or mechanisms related to the flexible implementation of task sets and related working memory processes (P3 ERP) do not contribute to cognitive inflexibility in IBD patients and were unchanged. It seems that pathophysiological processes in IBD strongly compromise cognitive-neurophysiological subprocesses related to fronto-striatal networks. These circuits may become overstrained in IBD when cognitive flexibility is required.

## Introduction

Inflammatory bowel disease (IBD) is a highly prevalent and lifelong relapsing disease with ulcerative colitis and Crohn’s disease being the best-studied representatives. In the last two decades, numerous studies focused on the pathophysiology of the intestinal mucosa as well as on the reduction of recurrent inflammatory flares^1–3^. This is of great importance, as incidences are growing worldwide causing enormous demands upon healthcare resources. However, despite the interplay between brain and gut is well established^4^, little knowledge exists in how far this pathophysiology of IBD affects cognitive functions, especially when it comes to the underlying neurophysiological mechanisms.

Regarding cognitive processes, especially executive control functions are important as these are known to impact daily life competencies^5^. One aspect of executive control is the ability to flexibly switch behavior upon changes in the environment^5^. However, such task switching abilities and mechanisms subserving cognitive flexibility are closely intertwined with other cognitive functions like working memory processes^6^. Within the context of the pathophysiology of IBD, these processes may be particularly relevant. This is because processes important for task switching and cognitive flexibility strongly depend on basal ganglia-prefrontal cortical circuits^7–10^. In these circuits, the tumor necrosis factor alpha (TNF-α) is known to compromise neural processes and induces neurodegenerative effects^11–14^. Since TNF-α is strongly elevated in IBD and plays a central role in the pathophysiology of IBD^15^ including the increased synthesis of cytokines within the central nervous system^16,17^, it is likely that task switching and cognitive flexibility processes are compromised in IBD.

In the current study we examine task switching processes in IBD with focus on the neurophysiological mechanisms and associated functional neuroanatomical networks being altered. Using event-related potentials (ERPs) different cognitive-neurophysiological subprocesses involved during information processing can be isolated on the basis of their temporal occurrence. In combination with source localization techniques this makes it possible to examine changes in the functional neuroanatomical network.

Switching between responses leads to an increase in processing times^18^. These switch costs represent an additional active reconfiguration process^18^, or interference from the previous trial involving different cognitive subprocesses related to response selection and the resolution of conflict, the inhibition of irrelevant task sets and retrieving goals and rules from working memory^19–21^. On a neurophysiological level, such processes are dissociable in the N2 and P3 ERPs. The N2 is considered to reflect increased response selection and the resolution of conflict^10,20,21^, while the P3 is considered to reflect the implementation of a switching task-set (P3 ERP)^19–27^. Interestingly, it has been shown that especially processes reflected by the N2 in medial frontal cortical structures are affected by modulations in TNF-α^8,28^ and hence a major factor in the pathophysiology of IBD. It is therefore possible that specifically medial frontal response selection processes during task switching are dysfunctional in IBD and that little or no modulatory effects are evident in other cognitive-neurophysiological subprocesses involved in task switching - i.e. task set implementation processes reflected by the P3. However, as outlined above task switching abilities and mechanisms subserving cognitive flexibility are strongly modulated by working memory processes^6,10,29^. Since increasing demands on working memory processes has been shown to compromise switching performance, it is possible that IBD patients show strongest deficits in switching when working memory load is increased. However, since working memory load during task switching does not specifically modulate cognitive subprocesses reflected by the N2 and medial frontal cortices^30^ it is possible that working memory load does not further complicate task switching in IBD patients. The entire hypothesized pattern of results would suggest that even though the pathophysiology of IBD is diverse, the mechanisms leading to cognitive dysfunction and cognitive inflexibility in particular are quite specific. However, since perceptual gating and attentional selection processes, reflected by the P1 and N1 ERP^31^, have been shown to be modulated by TNF-α^8,32,33^ we explore in how these processes are changed in IBD and contribute to altered cognitive flexibility in IBD.

## Results

### Behavioral data

#### Accuracy

A mixed effects ANOVA revealed a significant main effect for the factor “repetition-switch” was detected (*F*_1,42_ = 116.77; *p* < 0.001; *η* ^2^ = 0.307). Accuracy for repeated responses was higher (94.85 ± 0.69) than for switched responses (93.22 ± 0.77). Moreover, significant interaction of “repetition-switch × group” (*F*_1,42_ = 6.27; *p* = 0.016; *η* ^2^ = 0.130). For the post-hoc tests, the switch costs were calculated (i.e. switch minus repetition). The switch costs were higher in the IBD patients (2.59 ± 3.0) than in the control group (0.69 ± 2.01) (*t*_42_ = 2.50; *p* = 0.016). No other effects were significant (all *F* < 2.40; *p* > 0.1). Further analyses were conducted comparing the control group and only those patients, which were treated with TNF-α blockers. The mixed effects ANOVA revealed a significant main effect for the factor “repetition-switch” (*F*_1,29_ = 12.52; *p* < 0.001; *η* ^2^ = 0.302). Accuracy for repeated responses was higher (95.43 ± 1.04) than for switched responses (93.57 ± 1.12). Additionally, the significant interaction of “repetition-switch x group” (*F*_1,29_ = 4.99; *p* = 0.033; *η* ^2^ = 0.147) was replicated. Post-hoc tests again endorsed higher switch costs in the IBD group (3.03 ± 3.66) than in the control group (0.69 ± 2.01) (*t*_29_ = 2.23; *p* = 0.033). Thus, the results were similar to the results of the whole group of patients.

#### Response times

A mixed-effects ANOVA revealed a significant main effect for “block (cue vs. memory)” (*F*_1,42_ = 9.23; *p* = 0.004; *η* ^2^ = 0.180) with faster responses during the cued block (751 ± 26) than during the memory-based block (788 ± 22). Additionally, a main effect “repetition-switch” was detected (*F*_1,42_ = 52.26; *p* < 0.001; *η* ^2^ = 0.554). RTs on repeated responses (735 ± 22) were faster than RTs on switched responses (804 ± 25). Furthermore, an interaction of “repetition-switch x group” (*F*_1,42_ = 5.02; *p* = 0.030; *η* ^2^ = 0.107) was revealed. The switch costs (calculated as switch minus repetition) were higher in IBD patients (91.14 ± 80.15) than in controls (48 ± 45.52) (*t*_29_ = 2.14; *p* = 0.041). Finally, a significant interaction of “repetition-switch x block (cue vs. memory)” was shown (*F*_1,42_ = 27.31; *p < *0.001, *η* ^2^ = 0.394) with greater switch costs during the memory-based block (96 ± 77) than in the cue-based block (38 ± 73). This was confirmed by the post-hoc test (*t*_43_ = 5.33; *p* < 0.001). No other effects were significant (all *F* < 1.99, *p* > 0.1).

As for the accuracy results, we also compared the control group and only those patients, which were treated with TNF-α blockers. The mixed-effects ANOVA revealed the significant main effect for “repetition-switch” (*F*_1,29_ = 32.04; *p* < 0.00; *η* ^2^ = 0.525) with faster responses during repeated trials (742 ± 33) than during switched trials (823 ± 39). Furthermore, a significant interaction of “repetition-switch x group” (*F*_1,29_ = 5.36; *p* = 0.028; *η* ^2^ = 0.156) was shown. The post-hoc tests replicated the higher switch costs in the IBD group (114 ± 116) compared to the control group (46 ± 45) (*t*_28_ = 2.32; *p* = 0.028). Finally, the significant interaction of “repetition-switch x block (cue vs. memory) was replicated (*F*_1,29_ = 5.88; *p* = 0.022; *η* ^2^ = 0.169). Switch costs were higher in the memory-based block (86 ± 77) than in the cue-based block (39 ± 79) (*t*_30_ = 3.97; *p* < 0.001).

### Neurophysiological data

#### Attentional selection processes

**Figure 1:**
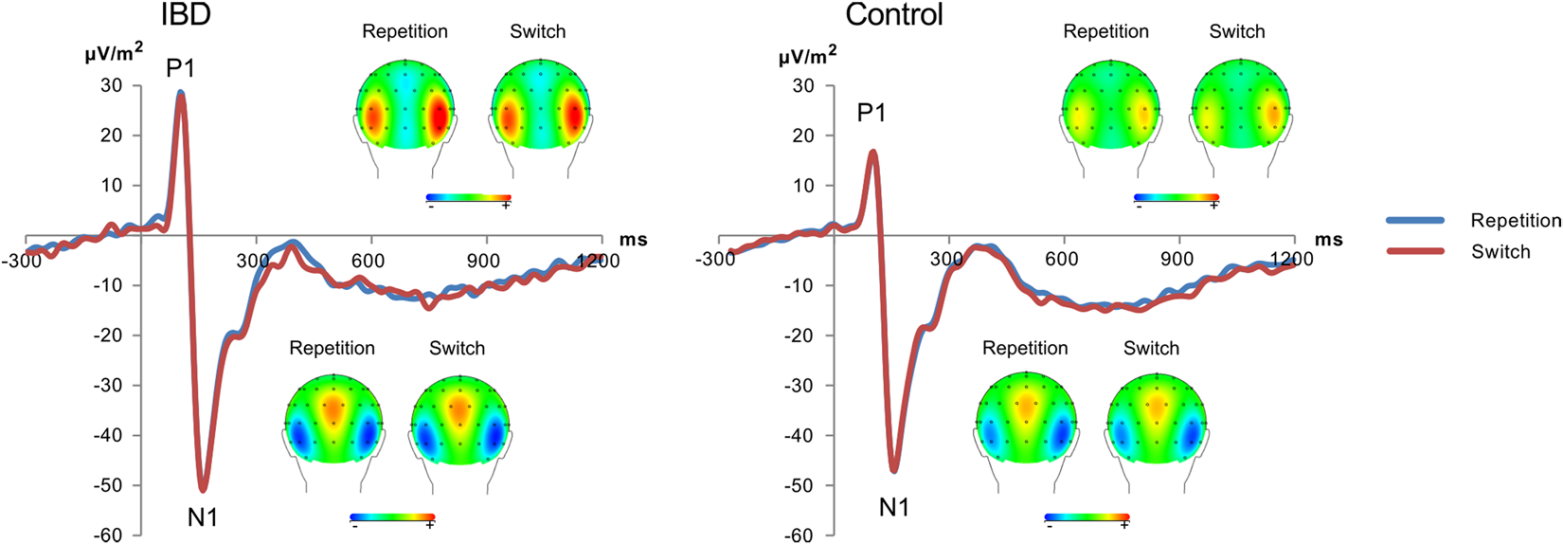
Event-related potentials for IBD patients (left) and controls (right) showing the P1 and N1 on the repeat (blue) and switch trials (red) pooled across electrode P9 and P10. Time point zero denotes the time point of target stimulus presentation. The scalp topography plots show clear P1 and N1 topographies at the peak of the respective ERP component for switch and repetition trials.

The P1 and N1 data are shown in Fig. 1. A significant main effect of the factor “block” was given for the P1 amplitudes (*F*_1,42_ = 11.90; *p* = 0.001; *η* ^2^ = 0.221) showing that amplitudes were higher in the memory-based block (23.81 µV/m^2^ ± 2.39) than in the cue-based block (21.08 µV/m^2^ ± 2.17). Additionally, a main effect “electrodes” was detected (*F*_1,42_ = 6.07; *p* = 0.018; *η* ^2^ = 0.126) with higher amplitudes at electrode P10 (25.73 µV/m^2^ ± 3.29) than at electrode P9 (19.15 µV/m^2^ ± 1.68). No other effects were significant (all *F* < 2.06, *p* > 0.1).

Analysis of the N1 showed a main effect for “block” (*F*_1,42_ = 32.85; *p* < 0.001, *η* ^2^ = 0.439). Analogue to the P1 results, amplitudes for the memory-based responses were higher (−52.04 µV/m^2^ ± 4.27) than for the cue-based responses (−45.21 µV/m^2^ ± 3.93). Moreover, a significant interaction of “block x electrode” was detected (*F*_1,42_ = 4.80; *p* = 0.034; *η* ^2^ = 0.103). Post-hoc tests were calculated for amplitude differences (P9 minus P10) between the blocks. A paired samples t-test revealed a higher amplitude difference for cue-based responses (7.55 µV/m^2^ ± 8.93) compared with memory-based responses (5.69 µV/m^2^ ± 8.17) (*t*_43_ = 2.27, *p* = 0.028). No other effects were significant (all *F* < 1.95; *p* > 0.1).

#### Response selection processes

**Figure 2:**
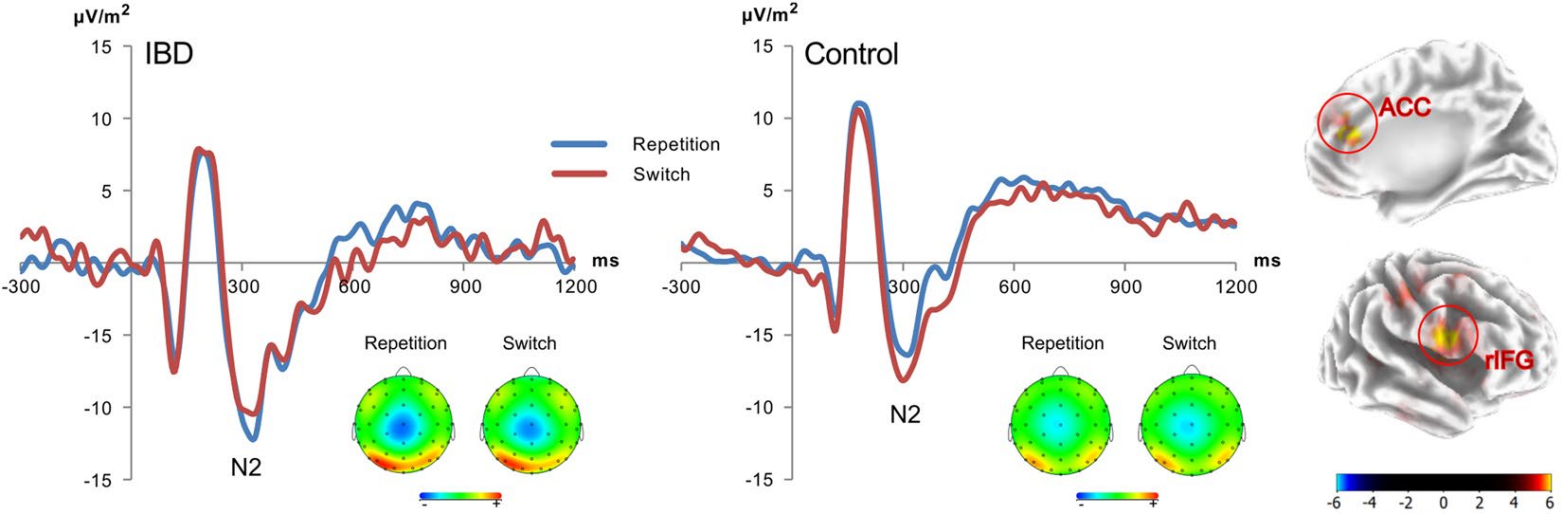
Event-related potentials for IBD patients (left) and controls (right) showing the N2 on repeat (blue) and switch trials (red). Time point zero denotes the time point of target stimulus presentation. The scalp topography show a typical negativity centered on electrode Cz. The sLORETA plot shows the source of the switch cost difference between the groups. Activation differences in the anterior cingulate cortex (ACC) and the right inferior frontal gyrus (rIFG) (BA 24 and BA 44/45) are shown. The control group shows a higher activation than the IBD group. The sLORETA colour scale indicates critical t-values corrected for multiple comparisons.

**Figure 3:**
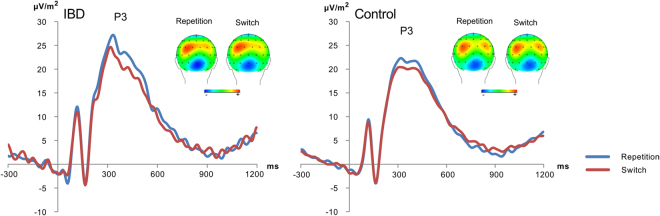
Event-related potentials for IBD patients (left) and controls (right) showing the P3 on repeat (blue) and switch trials (red). Time point zero denotes the time point of target stimulus presentation. The scalp topography plots show clear P3 topographies at the peak of the respective ERP component for switch and repetition trials.

The N2 ERP component is shown in Fig. 2 and the P3 ERP component is shown in Fig. 3. For the N2 amplitudes, the mixed-effects ANOVA revealed a significant interaction of “repetition-switch × group” (*F*_1,42_ = 4.33; *p* = 0.044; *η* ^2^ = 0.093). For the post-hoc tests, amplitude differences were calculated (repeated minus switched). Differences were positive in the group of controls (1.70µV/m^2^ ± 4.01) and negative in IBD patients (−1.71 µV/m^2^ ± 6.72). This means that controls showed higher N2 amplitudes for switched responses compared to repeated responses, while IBD patients displayed the opposite effect. The results of the post-hoc test underlines the difference between the groups (*t*_29_ = 1.99; *p* = .028). The sLORETA analysis, corrected for multiple comparisons (*p* < .01), shows that these differential modulations between groups were due to activation differences in the anterior cingulate cortex (ACC) and the right inferior frontal gyrus (rIFG) (controls > IBD patients). Furthermore, for the N2 amplitudes, an interaction of “repetition-switch x block” was detected (*F*_1,42_ = 78.15; *p* = .044; *η* ^2^ = .093). Amplitude differences between repeated and switched responses were calculated for each block (switch minus repetition). For the cue-based block a negative difference was found, which indicates higher amplitudes during repeated than switched responses (−1.13 ± 7.21). Conversely, in the memory-based block the difference was positive (1.43 ± 6.89), which indicates higher amplitudes during switched compared to repeated responses. Post-hoc tests confirmed the difference between the blocks (*t*_43_ = 1.99; *p* = .027). No other effects were detected (all *F* < 3.11; *p* > .1).

For the P3, main effects for “block” (*F*_1,42_ = 7.86; *p* = .008; *η* ^2^ = .158) and “repetition-switch” (*F*_1,42_ = 24.45, *p* < .001, *η* ^2^ = .368) were obtained. In particular, P3 amplitudes were larger in the memory-based (21.75 µV/m^2^ ± 1.29) than the cue-based block (20.38 µV/m^2^ ± 1.29), and during repeated (22.05 µV/m^2^ ± 1.30) than switched responses (20.09 µV/m^2^ ± 1.26). No other effects were detected (all *F* < 2.07; *p* > 0.1).

#### *Validation of the lack of findings of P1*, *N1 and P*3 *ERPs*

Above results suggest that only in some cognitive subprocesses differences between IBD patients and controls were evident. To validate the lack of effect observed in some cognitive neurophysiological processes (i.e. to verify the null hypothesis), additional Bayesian analyses as proposed by Wagenmakers^34^ were conducted using the method by Masson^35^ to calculate the probability of the null hypothesis being true given the data p(H_0_/D). According to Raftery^36^, values higher 0.5 indicate that the null hypothesis is more likely to be true than the alternative hypothesis. For the interaction of repetition-switch × group, we calculated a probability of p(H_0_/D) = 0.86 for the N1 ERP, of p(H_0_/D) = 0.81 for the P1 ERP and of p(H_0_/D) = 0.70 for the P3 ERP. These additional results provide very strong evidence in favor of the null hypothesis. Since the sample consisted of patients suffering from Crohn’s disease (CD) or ulcerative colitis (UC) we also examined in how far there are differences between Crohn’s disease (CD) patients and controls, as well as differences between ulcerative colitis (UC) patients and controls; i.e. we perform a subgroup analysis, because it is well documented that CD considerably differs from UC regarding clinical, pathological, and biomolecular features^37^. This analysis can be found in the supplementary analysis.

## Discussion

Inflammatory bowel diseases (IBD) show world-wide increasing incidences^38,39^ and knowledge on the treatment and pathophysiology has made considerable improvements in the last years. However, while there has also been increasing urgency to understand cognitive disturbances associated with IBD^40,41^, these changes are far from being understood, especially when it comes to the neurophysiological mechanisms behind these changes.

Executive control functions are known to impact daily life competencies^5^. However, important subprocesses of executive control functions like working memory processes and mechanisms subserving cognitive flexibility are closely intertwined in daily life^6^. In the current study we therefore examined how cognitive flexibility processes and their known dependence on working memory processes are modulated in IBD and what neurophysiological (EEG) mechanisms underlie these changes. The results reported are robust since also a subgroup analysis (refer supplemtary analysis) comparing ulcerative colitis patients against controls and comparing Crohn’s disease patients against controls revealed the same pattern of results.

On a behavioral level, both groups displayed switch costs showing lower accuracies and slower responses in switch trials than in repeated trials^30,42,43^. Importantly, IBD patients showed higher switch costs than controls. This effect was not differentially modulated between the cue-based block and the memory-based block, suggesting that working memory processes are not critical to consider for dysfunctions in cognitive flexibility in IBD patients. As a corollary, this suggests that pathophysiological processes in IBD do not affect neurobiological systems and functional neuroanatomical structures that subserve working memory processes to an extent that it affects cognitive flexibility. Rather, neural mechanisms subserving switching and cognitive flexibility are predominantly affected.

The neurophysiological data showed that changes associated with dysfunctions in cognitive flexibility affected very specific cognitive subprocesses. The N1 ERPs, for example, did not show interactive effects between “group” and “repetition-switch” suggesting that bottom-up attentional selection processes (reflected by the N1)^31^ do not contribute to cognitive inflexibility. Similarly, the P1 did also not show interactive effects. The P1 is often considered to reflect simple processes of perceptual gating^31,44^, but may also reflect early stimulus categorization processes^45,46^ needed to filter out relevant stimulus features in task relevant networks and to block information processing in potentially competing task irrelevant networks^45^. However, these processes were not modulated between groups and cognitive subprocesses and are therefore unlikely to be affected by pathophysiological changes occurring in IBD. Also, the P3 ERP did not show differential group effects between repetition and switch trials. This suggests that processes related to the implementation of a switching task-set (P3 ERP)^19–27^ are unlikely to be the mechanisms behind cognitive inflexibility in IBD. These interpretations are supported by the bayesian analyses providing strong evidence for the null hypothesis, a lack of effects in IBD patients.

However, differential group effects between switch and repetition trials were observed for the N2 ERP. The control group revealed the usual increase in N2 amplitude in switch, compared to repetition trials^43,47–50^. These increases have been suggested to reflect increased response selection and the resolution of conflict^19–21^. These conflicts emerge because the previous task-set (rule) is still active and needs to be replaced by the new task-set. Importantly, IBD patients did not show an increase in N2 amplitudes on switch trials. Rather, the N2 showed a relative decrease in switch, compared to repetition trials. In combination with the behavioral data pattern, this suggests that response selection processes cannot appropriately be activated when a new task set needs to be implemented. It is not the processes of the implementation or updating of the task set per se, which is reflected by the P3 not modulated in IBD patients, but the process of response selection. The source localization analysis shows that activation differences in the medial frontal cortex and especially the anterior cingulate cortex (ACC), as well as the right inferior frontal gyrus (rIFG) were related to modulations in the N2 between IBD patients and controls. It thus seems that pathophysiological processes in IBD affect medial frontal and right inferior frontal areas, which affects cognitive flexibility processes because response selection mechanisms are reduced in their efficiency. Since switching involves inhibition processes of irrelevant task sets during conflict monitoring and response selection^18,43,51,52^, it is possible that these are reflected in activation differences in the rIFG. The rIFG has frequently been shown to be involved in such inhibitory control processes^53^.

A major inflammatory mechanism in IBD relates to increased TNF-α activity^17^, which is therefore targeted in treatments^54,55^. In the brain, TNF-α is well known to be able to exert neurotoxic and neuroprotective effects, depending on differential patterns of expression of TNF receptors on neuronal cells^56,57^. A number of studies have shown that especially in the basal ganglia TNF-α can induce and is a key-player of striatal (dopaminergic) neurodegeneration^11,13,14^. Importantly, the ACC and rIFG show strong functional connections with the basal ganglia^9^. It is therefore possible that the findings in this study reflect an effect of neurotoxic effects of TNF-α at the striatal level that compromise response selection mechanisms in the ACC and rIFG. This interpretation is in line with several other lines of evidence: First, it has been shown that dysfunctions at the basal ganglia level affect response selection processes reflected by the N2 ERP^58^, possibly by affecting up-stream medial frontal cortical processes^59–61^. Second, several studies have shown that TNF-α modulates cognitive control and flexibility processes^8,28,32,33^ and it has been shown that especially response selection processes reflected by the N2 ERP are affected by modulations in TNF-α^7,28^. These lines of evidence make it likely that dysfunctions in cognitive flexibility in IBD emerge because response selection mechanisms become overstrained, since inflammatory processes in IBD compromise fronto-striatal networks important for response selection mechanisms. This interpretation is further supported by the results of our additional analyses of the control group and patients treated with TNF-α blockers. Despite the self-reported disease conditions, medication treatments were very heterogeneous within both, patients experiencing actual flares and patients in remission. This means, all patients were currently, or had been affected by elevations in TNF-α during the course of their disease.

As regards the study’s limitation, it must be mentioned that the sample has been relatively small. Moreover, the heterogeneous medication profile in the IBD group is also a limitation of the study. Yet, the effect sizes obtained and also the results from the bayesian analysis show that the results obtained are robust. The study’s limitations do therefore not critically affect the validity of the results. Moreover, controlling the effects of depressive symptoms did not affect the pattern of results. Another limitation is that no reliable data is available regarding the onset of the diseases, but there are still no reliable questionnaire available that examines the disease course and onset of the very much fluctuating IBD diseases. Existing measures like the German Inflammatory Bowel Disease activity Index (GIBDI CD/UC) are very dependent on the subjective perception of the patient. It is therefore not possible to examine any reliable relation of disease duration and the processes we examined in our study. Future studies should focus more on the discriminability of the current state of the respective disease, for example by performing a prompt colonoscopy in order to determine the current status of the symptoms. Future studies may also include a standard neuropsychological assessment. While this may be regarded as a limitation of this study it is important to note that such assessments can mostly detect more severe dysfunctions. Moreover, the results of the current study clearly show that deficits in IBD patients are not driven by possible working memory problems and the neurophysiological data also suggests that attentional selection mechanisms are also not affected in IBD. To examine the neurobiological mechanisms that may have led to cognitive flexibility changes in IBD, future study may use animal models.

In summary, this is the first study providing a detailed examination of cognitive flexibility processes in IBD including the associated neurophysiological processes. The results show that there are task switching deficits (i.e. increased switch costs) in IBD patients. The neurophysiological data show that even though the pathophysiology of IBD is diverse and wide-spread, only specific cognitive subprocesses are altered. There was a selective dysfunction at the response selection level (N2 ERP) associated with functional alterations in the anterior cingulate cortex and the right inferior frontal gyrus. Attentional selection processes (N1 ERP), perceptual categorization processes (P1 ERP), or mechanisms related to the flexible implementation of task sets and related working memory processes (P3 ERP) do not contribute to cognitive inflexibility in IBD patients and were unchanged. It seems that pathophysiological processes in IBD strongly compromise cognitive-neurophysiological subprocesses related to fronto-striatal networks. These mechanisms likely become overstrained when cognitive flexibility is required.

## Materials and Methods

### Participants

The sample consisted of 46 participants between 19 and 30 years (M = 25.57; SD = 2.82). 2 participants had to be excluded because of poor data quality of the EEG recordings. N = 20 of the remaining participants (M = 25.35; SD = 3.07) suffered from IBD, i.e. Crohn’s disease and ulcerative colitis and were recruited locally in the clinic of gastroenterology department TU Dresden after clinical diagnosis by a physician. Diagnosis of IBD was required to meet the European Crohn’s and Colitis Organization criteria^62,63^. 24 participants (M = 25.67; SD = 2.73) served as a control group. All participants had normal or corrected-to-normal vision. Demographical and clinical details of the patients and controls are shown in Supplementary Table 1.

Prior to the measurements, participants gave written informed consent and were treated according to the declaration of Helsinki. Participants subsequently filled in questionnaires about their demographics and self-reported psychological well-being. This included the german versions of the “Beck Depression Inventory (BDI)”^64^ and the “Fatigue Scale for Motor and Cognitive Functions (FSMC)”^65^. We also conducted the “Multiple Choice Word Test-B” (MWT-B)^66^, which is a valid and short test to estimate premorbid intelligence. No differences were found between the groups (p = .47). After testing, participants received a reasonable expense allowance of 30 Euro. The study and all included procedures were approved by the ethics committee of the TU Dresden.

### Stimuli and task

A published task switching paradigm established by our group was used in this study^29,30^ to examine cognitive flexibility and the possible modulation by working memory processes. At the beginning of the experiment participants were given instructions and requested to conduct an exercise for 18 trials to become familiar with the stimuli and task-rules. All participants were encouraged to answer as quick and accurate as possible.

**Figure 4:**
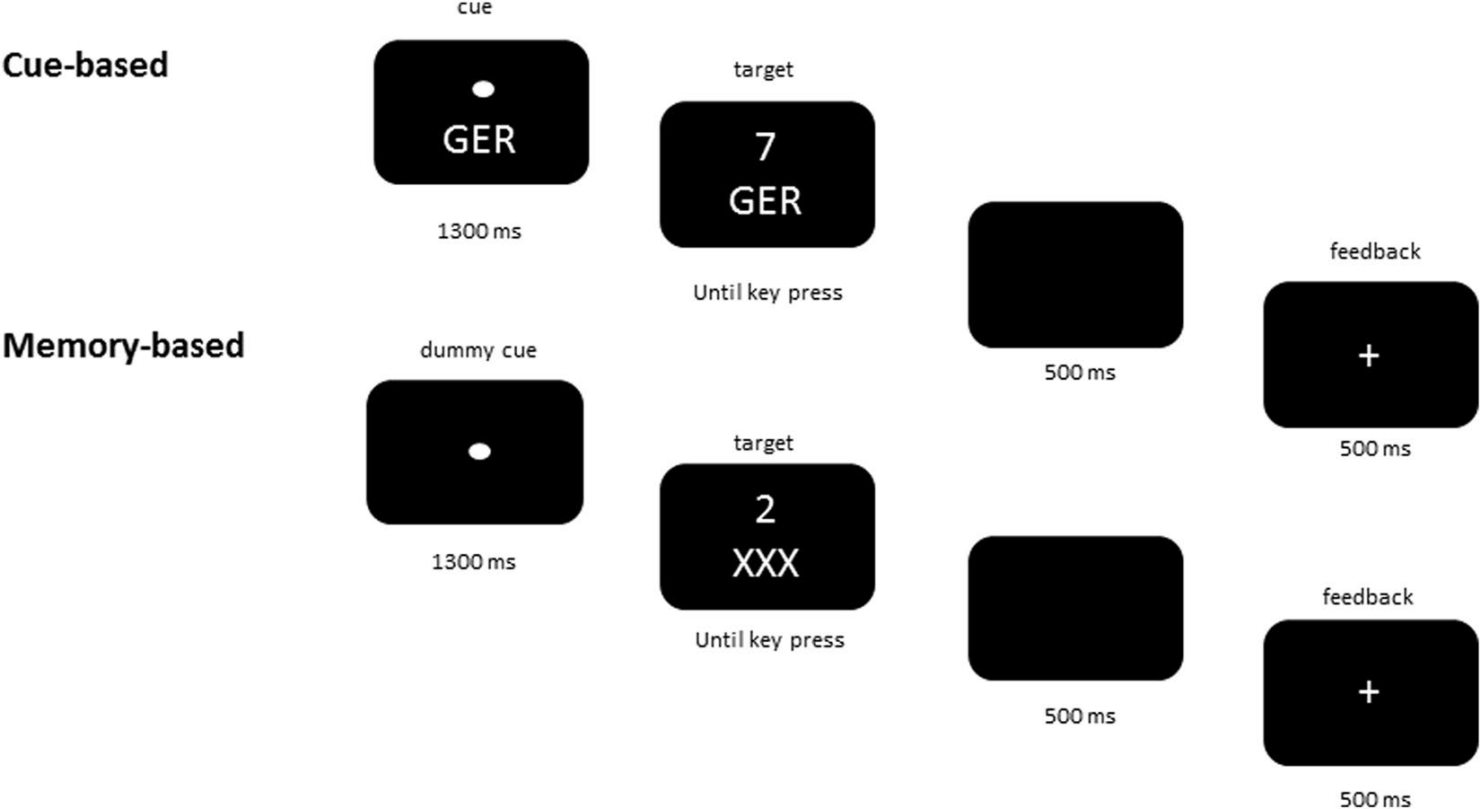
Schematic illustration of the switch paradigm. The figure shows (**a**) one trial for the cue-based condition and (**b**) one trial for the memory-based condition. Figure taken from N. Wolff *et al*.^29^.

The task consisted of digits from 1-9, with the exclusion of the number 5, presented in white on a black background on a 20 inch CRT monitor. The digits were presented 3mm above the white fixation cross of 10mm diameter in two different sizes for the characterization of different conditions of subsequent tasks; small (7 × 10mm) and large (12 × 18mm). An explicit task cue, which indicated the corresponding task rule, was presented 3mm below the fixation cross. Three explicit task cues were relevant for the performance. Cue “NUM” (Ger. abbreviation for “Numerisch”, Eng. “numeric”) stood for the question “Is the presented digit smaller or greater than the number 5?”. Cue “GER” (Ger. abbreviation for “Geradzahligkeit”, Eng. “parity”) stood for the question “Is the presented digit odd or even?”. The last cue “SG” (Ger. abbreviation for “Schriftgröße”, Eng. “font-size”) asked for the question “Is the digit presented in small or large font-size?”. The entire task consisted of two blocks – a cued block and a memory block. These explicit task cues were only presented in the cued block. In the memory block a dummy cue “XXX” was presented in every trial instead of the informative cues (3mm below the fixation cross) to keep the visual setup of the task comparable. Responses were given by pressing left or right control key (Ctrl buttons) of a standard PC keyboard. The left control had to be pressed for responses indicating “smaller than 5”, “odd number”, and “small font-size” and the right control key for the respective contrary responses. A schematic example of both, a trial in the cue-based task and the memory-based task is presented in Fig. 4.

The two different blocks were tested consecutively. The cue-based task was presented first, followed by the memory-based task block. In the cue-based block, a trial always began with the presentation of the fixation point. Then, one of the three possible explicit task cues was presented for 1300 ms and remained visible during the target (digit) presentation. After target-onset, participants were asked to respond as fast and accurately as possible within a time interval of 2500 ms. Once the response had been executed a feedback stimulus was displayed with a latency of 500 ms and had duration of 500 ms. Feedback for a correct response was indicated by a plus sign, incorrect responses were displayed by a minus sign. 300 ms thereafter, the feedback stimulus disappeared and the next explicit task cue was presented. Cue stimuli were shown in randomized order and forced participants to repeat or switch stimulus-response task-sets. Maximally four consecutive task-repetitions were presented. The task consisted of 198 trials. After every 33 trials, participants were able to take a quick break and continue the next trial themselves by pressing one of the control keys. In the memory-block participants had to execute the same task with the following deviations. Instead of following the explicit task cue at the beginning of every trial, participants were instructed to follow the same run of tasks AAABBBCCC (3 × NUM, 3 × GER, and 3 × SG) for the whole task. Additionally, the explicit task cues were replaced with the uninformative dummy cue “XXX” in every trial. Thus, participants were obliged to keep the trial sequence in mind. In case of three consecutive incorrect, or not given responses within the 2500 ms time interval, written rule instructions were presented on the screen and at this time point explicit task cues were given for the next three trials. In doing so, participants were helped getting back on track. The memory block also consisted of 198 trials. Again, after every 33 trials, participants were given the chance to pause and decide when to start with the next 33 trials.

### EEG recordings and data processing

The electroencephalogram (EEG) was recorded continuously from 60 Ag/AgCl scalp electrodes in equidistant positions. The ground electrode was positioned at coordinates *θ* = 58, *φ* = 78 and the reference electrode was positioned at coordinates *θ* = 90, *φ* = 90. The sampling rate was 500 Hz. All electrode impedances were kept below 5 kΩ. First, the data preprocessing included a manual inspection, where visible technical artifacts were removed. Afterwards, a band-pass filter was applied (0.5 to 20 Hz, slope of 48db/oct) and the data was down-sampled to 256 Hz. Next, an independent component analysis (ICA) was applied to remove recurring artifacts like eye movements, blinks and pulse artifacts. After discarding the corresponding components, the EEG was reconstructed. Then, target-locked segments (1300 ms after cue stimulus presentation) were built for all trials with correct responses. The overall segment length was 1500 ms, starting 300 ms prior to the locking time point and ended 1200 ms thereafter. An automated artifact rejection procedure then discarded all segments containing signal-amplitudes higher than 150 μV, lower than −150 μV, with periods of 200 ms showing lower activity than 0.5 μV, or amplitudes differences higher than 80 µV over a time interval of at least 100 ms. Then, data was re-referenced by using current source density (CSD) transformation. The CSD uses the potential difference between one electrode and the total potential of all surrounding electrodes to eliminate the reference potential (used parameters included n = 4 splines and m = 10 Legendre polynomials (Lambda = 1 × 10^−5^). Additionally, the procedure serves as spatial filter that helps identifying electrodes that can be analyzed for different ERP components^67^. Next, a baseline correction procedure was applied (−200 ms to 0) and averages were calculated for each condition (cue repetition, cue switch, memory-repetition, and memory-switch) at the single subject level before building group averages (controls vs. patients). According to the ERPs P1, N1, N2 and P3, electrodes were chosen visually after the scalp topography. The P1 and N1 were measured at electrodes P9 and P10 at 100–105 ms and 155–160 ms, respectively, the N2 was measured at electrode Cz at 295–330 ms and the P3 was measured at the P3 and P4 at 290–500 ms. Data were quantified at the single subject level. The choice of electrodes and time windows was statistically validated using the procedure described previously^68^.

For the source localization analyses sLORETA (standardized low resolution brain electromagnetic tomography^69^; was used, which provides a single solution to the inverse problem^69,70^. For sLORETA, the intracerebral volume is partitioned into 6239 voxels at 5 mm spatial resolution. Then, the standardized current density at each voxel is calculated in a realistic head based on the MNI152 template^71^. It has been mathematically proven that sLORETA provides reliable results without a localization bias^70^. Moreover, there is evidence from EEG/fMRI and neuronavigated EEG/TMS studies underlining the validity of the sources estimated using sLORETA^68,70^. The voxel-based sLORETA images were compared across conditions (repetition vs. switch) and experimental blocks (cue vs. memory-based block) using the sLORETA-built-in voxel-wise randomization tests with 2000 permutations, based on statistical nonparametric mapping (SnPM). Voxels with significant differences (p < .01, corrected for multiple comparisons) between contrasted conditions were located in the MNI-brain.

### Statistical analysis

The statistical analysis was carried out by IBM SPSS 23.0.0.3. The data were analyzed using mixed effects ANOVAs in which the factors “repetition/switch” and “block” (cue vs. memory) were included as within-subject factors and where “group” was included as between-subject factor. For the neurophysiological data the factor “electrode” was included as additional within-subject factor, if necessary. Greenhouse-Geisser correction was applied to all tests and post-hoc tests were Bonferroni-corrected. All variables included in the analyses were normally distributed.

### Ethical standards

The authors assert that all procedures contributing to this work have been conducted in accordance with the ethical standards of the relevant national and institutional committees on human experimentation and with the Helsinki Declaration of 1975, as revised in 2008. The study was approved by the local ethics committee of the Medical Faculty of the TU Dresden.

## Electronic supplementary material

Supplemental material
