# Supplementary material for: Specific neurophysiological mechanisms underlie cognitive inflexibility in inflammatory bowel disease
Source: Sci Rep. 2017 Oct 24;7:13943. doi: 10.1038/s41598-017-14345-5 (PMC5655331; doi:10.1038/s41598-017-14345-5)
Supplement: Supplementary file 1 — Supplemental material [file 41598_2017_14345_MOESM1_ESM.doc]

**Supplemental material**

**Specific neurophysiological mechanisms underlie cognitive inflexibility in inflammatory bowel disease**

Vanessa A Petruo^1,#^, Sebastian Zeißig^2^, Renate Schmelz^2^, Jochen Hampe^2^, Christian Beste^1,3^

**Supplemental analysis**

Our experiment showed behavioral differences between control participants and a very heterogeneous group of IBD patients. The sample consisted of patients suffering from Crohn’s disease or ulcerative colitis. In this supplementary analysis we examine in how far there are differences between Crohn’s disease (CD) patients and controls, as well as differences between ulcerative colitis (UC) patients and controls; i.e. we perform a subgroup analysis, because it is well documented that CD considerably differs from UC regarding clinical, pathological, and biomolecular features ^1^.

Mixed effects ANOVAs with the within-subject factors "repetition-switch" and "block" (cue vs. memory) and "group" (UC vs. controls, or CD vs. controls) as between-subject factor were calculated. For the analysis of the neurophysiological data, an additional within-subject factor “electrode” was included. The analyses revealed the same pattern of results as already described for the whole group of patients, namely higher switch-costs for the respective group of IBD patients. However, because of the smaller sample size in each subgroup, the statistical power is lower.

*Behavioral results for UC patients vs. controls*

With respect to the accuracy of responses in the group of UC patients, a significant main effect “repetition-switch” (*F*_1,32_=12.06; *p*= .001; *η^2^*= .274) revealed a higher accuracy on repetition (94.79 ±.94) than switch trials (93.33 ±1.06).

For the reaction times (RTs), a significant main effect for “block” (*F*_1,32_=6.59; *p*= .005; *η^2^*= .171) revealed faster responses in cued trials (766.85 ±32.21) than in memory trials (807.58 ±26.12). Furthermore, the main effect “repetition-switch” (*F*_1,32_=55.40; *p*< .000; *η^2^*= .634) showed that responses on repetition trials were faster (746.87 ±27) than responses on switch trials (827.53 ±30.39).Moreover, an interaction of “repetition-switch x group” was detected (*F*_1,32_=9.08; *p*= .005; *η^2^*= .221). Post-hoc switch costs were calculated (i.e. switch minus repetition). Consistently, the switch costs were higher in the UC patients (113.31 ±80.57) than in the control group (48 ±45.52) (*t*_32_=3.01; *p*= .005). Finally, a significant interaction of “repetition-switch x block” was evident (*F*_1,32_=23.54; *p*< .000; *η^2^*= .424) with higher switch costs during the memory-based block (97.02 ±73.09) than during the cue-based block (37.4 ±70.65) (*t*_33_=5.39; *p*< .001).

*Behavioral results for CD patients vs. controls*

Concerning “accuracy”, a main effect for the factor “repetition-switch” was shown (*F*_1,32_=15.19; *p*< .001; *η^2^*= .322) with higher accuracies for repeated trials (94.91 ± .89) than for switched trials (93.10 ± .92). Moreover, the mixed-effect ANOVA revealed a significant interaction of “repetition-switch x block x group” (*F*_1,32_=4.74; *p*= .037; *η^2^*= .129). Post-hoc tests showed higher switch-costs in the group of CD patients (3.85 ±4.12) compared to the controls ( .27±2.42) during the cue-based block (*t*_32_=3.18; *p*< .005), while switch-costs in the memory-based block, were not different between the groups (*t*_32_=0.81; *p>* .40). Moreover, a significant interaction of the factors “repetition-switch x group” was detected (*F*_1,32_=5.84; *p*= .022; *η^2^*= .154) with higher switch-costs for the CD patients (2.92 ±3.34) than for the controls ( .69 ±2.01) (*t*_32_=2.42; *p<* .03).

For the RTs, a main effect for the factor “block” was detected (*F*_1,32_=6.00; *p*= .020; *η^2^*= .158) with faster RTs in the cue-based block (736.33 ±35.29) than in the memory-based block (768.52 ±29.79). Moreover, a main effect for the factor “repetition-switch” was revealed (*F*_1,32_=30.45; *p*< .001; *η^2^*= .488) with faster responses for repeated trials (723.19 ±30.61) than for switched trials (781.67 ±34.14). Finally, an interaction of “block x repetition-switch” was shown (*F*_1,32_=14.63; *p*= .001; *η^2^*= .314). Post-hoc tests revealed higher switch-costs during the memory-based block (82.46 ±75.37) than during the cue-based block (25.86 ±58.83) (*t*_33_=4.41; *p<* .001). No other interaction effects with the group were detected (*F*_1,32_= .99; *p*> .30).

*Neurophysiological results for UC patients vs. controls*

A significant main effect for the factor “block” was given for the P1 amplitudes (*F*_1,32_=9.50; *p*< .005; *η^2^*= .229), showing higher amplitudes in the memory-based block (22.90µV/m^2^ ±2.78) than in the cue-based block (19.67 ±2.35). Moreover, a main effect “electrodes” was revealed (*F*_1,32_=17.07; *p*< .001; *η^2^*= .348) with higher amplitudes at electrode P10 (29.18µV/m^2^ ±3.95) than at electrode P9 (18.01µV/m^2^ ±1.99). Analogue to the P1 results, analysis of the N1 showed a main effect for “block” (*F*_1,32_=18.72; *p*< .001; *η^2^*= .369), again with higher amplitudes in the memory-based block (46.92µV/m^2^ ±5.11) than the cue-based block (40.58µV/m^2^ ±4.88). Furthermore, a main effect for the factor “electrodes” was given (*F*_1,32_=5.43; *p*< .03; *η^2^*= .145) with higher amplitudes at the P10 electrode (45.88µV/m^2^ ±5.96) than at the P9 electrode (41.62µV/m^2^ ±4.33).

For the N2 amplitudes, the mixed-effects ANOVA detected an interaction of “repetition-switch x group” (*F*_1,32_=6.70; *p*< .02; *η^2^*= .173). Post-hoc tests revealed higher switch costs for the UC patients (3.51µV/m^2^ ±7.78) than for the control group (1.7µV/m^2^ ±4.01) (*t*_32_=2.59; *p<* .02). Calculations for the P3 ERP revealed overall main effects for “block” (*F*_1,32_=10.30; *p*< .005; *η^2^*= .243) and “repetition-switch” (*F*_1,32_=23.22; *p*< .001; *η^2^*= .420). Amplitudes were larger for the memory-based block (22.85µV/m^2^ ±1.67) compared with the cue-based block (21.11µV/m^2^ ±1.75) and for repeated trials (23.19µV/m^2^ ±1.71) compared with switched trials (20.77µV/m^2^ ±1.70). Finally, an interaction effect of “repetition-switch x group” were shown (*F*_1,32_=4.21; *p*< .05; *η^2^*= .116). Post-hoc tests revealed higher amplitude differences for switch costs in the UC patients (3.45µV/m^2^ ±3.37) than in the controls (1.39µV/m^2^ ±2.34) (*t*_32_=2.05; *p<* .05).

*Neurophysiological results for CD patients vs. controls*

Calculations on the P1 amplitudes revealed an overall main effect for “block” (*F*_1,32_=6.17; *p*< .02; *η^2^*= .162) showing larger amplitudes in the memory-based block (24.70µV/m^2^ ±2.93) than in the cue-based block (22.49µV/m^2^ ±2.78). Additionally, a main effect for “electrodes” was given (*F*_1,32_=17.07; *p*< .001; *η^2^*= .348). Higher mean amplitudes were revealed at the P10 electrode (29.18µV/m^2^ ±3.95) than at the P9 electrode (18.01µV/m^2^ ±1.99). Regarding the N1 amplitudes, a main effect of “block” was detected (*F*_1,32_=21.73; *p*< .001; *η^2^*= .404) with higher amplitudes during the memory-based block (57.16µV/m^2^ ±5.51) than during the cue-based block (49.84µV/m^2^ ±5.00). This was also the case for the N2 ERP. An overall main effect for “block” (*F*_1,32_=5.18; *p*< .03; *η^2^*= .139) showed higher amplitudes during memory-based trials (7.92µV/m^2^ ±2.14) than during cue-based trials (5.72µV/m^2^ ±2.35). Additionally, an interaction of “block x repetition-switch” was shown (*F*_1,32_=6.75; *p*< .02; *η^2^*= .174). Post-hoc tests calculated higher amplitude differences for switch costs in the memory block (2.61µV/m^2^ ±6.38) compared to the cued block ( .15µV/m^2^ ±5.30) (*t*_33_=2.07; *p<* .05). Lastly, the P3 amplitudes detected an overall effect for the factor “repetition-switch” (*F*_1,32_=12.04; *p*< .005; *η^2^*= .273), revealing higher amplitudes for repeated responses (20.91µV/m^2^ ±1.69) compared to switched responses (19.41µV/m^2^ ±1.67). Moreover, a main effect for “electrodes” was shown (*F*_1,32_=5.02; *p*< .04; *η^2^*= .136) with higher amplitudes at the P9 electrode (22.90µV/m^2^ ±2.05) than at the P10 electrode (17.41µV/m^2^ ±2.09).

*References*

1. Rigoli, L. & Caruso, R. A. Inflammatory bowel disease in pediatric and adolescent patients: a biomolecular and histopathological review*. World J. Gastroentero*l**. 2**0, 10262–10278 (2014).

**Supplementary Table 1**

Demographic and psychological characteristics of the present sample, as well as medical treatments of IBD patients.

| N=44 | IBD (n=20) | Controls (n=24) |
| --- | --- | --- |
| Age, years (SD) | 25.4 (3.07) | 25.7 (2.73) |
| Gender, female | 45% | 58% |
| Disease, CD (UC) | 10 (10) | - |
| Academical education | 70% | 88% |
| In a Relationship | 10% | 12.5% |
| In Remission | 65% | - |
| BDI, mean (SD)  No symptoms  Mild depression  Moderate depression | 9.28 (7.15)  50%  40%  10% | 3.94 (3.85)  91.7%  8.3%  - |
| Smoker | 15% | 12.5% |
| BMI, mean (SD)  normal  slight preponderance | 22.4 (1.93)  90%  10% | 25.1 (6.28)  75%  25% |
| Fatigue, mean (SD)  No symptoms  Mild  Moderate  Severe | 53.85 (12.68)  20%  25%  25%  30% | 34.5 (9.79)  79.2%  16.7%  4.1%  - |
| Medication  TNF α-blockers  Cortison  Entyvio  Mesalazin  Azathioprin | 35%  30%  25%  50%  40% |  |
